# SARS-CoV-2 IgG Levels Allow Predicting the Optimal Time Span of Convalescent Plasma Donor Suitability

**DOI:** 10.3390/diagnostics12112567

**Published:** 2022-10-22

**Authors:** Sandra Laner-Plamberger, Nadja Lindlbauer, Lisa Weidner, Simon Gänsdorfer, Lukas Weseslindtner, Nina Held, Wanda Lauth, Georg Zimmermann, Jan Marco Kern, Fabian Föttinger, Laura Ombres, Christof Jungbauer, Eva Rohde, Christoph Grabmer

**Affiliations:** 1 Department for Transfusion Medicine, University Hospital of Salzburg, Paracelsus Medical University Salzburg, Müllner-Hauptstraße 48, 5020 Salzburg, Austria; 2 Austrian Red Cross, Blood Service for Vienna, Lower Austria and Burgenland, Wiedner Hauptstraße 32, 1040 Vienna, Austria; 3 Department for Virology, Medical University Vienna, Kinderspitalgasse 15, 1090 Vienna, Austria; 4 Team Biostatistics and Big Medical Data, IDA Lab Salzburg, Paracelsus Medical University Salzburg, Strubergasse 16, 5020 Salzburg, Austria; 5 Research and Innovation Management, Paracelsus Medical University Salzburg, Strubergasse 16, 5020 Salzburg, Austria; 6 Department for Clinical Microbiology and Hygiene, University Hospital of Salzburg, Paracelsus Medical University Salzburg, Müllner-Hauptstraße 48, 5020 Salzburg, Austria; 7 GMP Unit, Spinal Cord Injury & Tissue Regeneration Centre Salzburg (SCI-TReCS), Paracelsus Medical University, 5020 Salzburg, Austria

**Keywords:** plasmapheresis, convalescent plasma, COVID-19, SARS-CoV-2 antibodies

## Abstract

Convalescent plasma (CP) has been in use for the treatment of numerous infectious diseases for more than a century, recently also for coronavirus disease 2019 (COVID-19). A major challenge for this treatment is identifying suitable donors with sufficient levels of functional antibodies and to determine the optimal time span for CP donation. In this retrospective study, we analyzed 189 CP donations of 66 donors regarding anti-SARS-CoV-2 anti-S IgG antibody levels. We found a significant correlation between the semi-quantitative SARS-CoV-2 IgG ratio values and in vitro antibody functionality. A time-to-event analysis allowed us to predict the optimal time span of COVID-19 CP donor suitability. We found that high IgG ratio values, which significantly correlate with high in vitro antibody functionality, were suitable for CP donation for a median of 134 days after the first CP donation. Donors with lower IgG ratios were suitable for a median of 53 days. Our data support plasma collection centers to determine optimal points in time for CP donation by means of widely used semi-quantitative laboratory IgG ratio values.

## 1. Introduction

Since 2019, COVID-19, caused by severe acute respiratory syndrome coronavirus-2 (SARS-CoV-2), is endangering global health and the economy. Despite astonishing achievements regarding diagnostics [1,2] and vaccination [3,4,5] within a very short time period, efficient and safe treatment options particularly for critically ill COVID-19 patients are still rare. Frequently corticosteroids such as dexamethasone are applied, which are used to treat COVID-19- and non-COVID-19-induced acute respiratory distress syndrome (ARDS) because of its anti-inflammatory and immunosuppressive effects [6,7]. Since the end of 2021, the combination of the antiviral protease inhibitors ritonavir and nirmatrelvir, which allow for impeding viral replication, is applied for the treatment of severely affected COVID-19 patients [8,9,10]. This seemed to be a very promising strategy to fight severe health damage. However, there are reports about cases of COVID-19 relapse after the end of the antiviral treatment [11,12]. In addition, further studies recently report resistance mutations in SARS-CoV-2’s protease against these antiviral drugs [13,14].

Another therapeutic strategy to treat COVID-19 patients is the administration of convalescent plasma (CP). Convalescent plasma therapy, which passively immunizes patients by the transfer of specific, neutralizing antibodies from recovered individuals, has been in use for more than 100 years to treat different infectious diseases such as Spanish influenza, poliomyelitis, measles, mumps, hepatitis A and B and, more recently, SARS, Ebola virus disease and influenza A [15,16]. Despite contradictory results concerning the efficacy of this therapy form when treating severely affected COVID-19 patients [17,18,19,20,21], many countries collect and administer CP, as there are data indicating that CP is a safe and efficient therapy, providing that plasma with sufficient anti-SARS-CoV-2 antibody levels is administered early in the disease course [22,23].

Identifying potential SARS-CoV-2 CP donors is still difficult due to varying specific antibody levels among recovered and/or vaccinated individuals. Furthermore, different laboratories use various test systems and methods to determine the levels of different types of anti-SARS-CoV-2 antibodies. However, the traditional and commonly applied enzyme-linked immunosorbent assays (ELISA) or electrochemiluminescence immunoassays (ECLIA) detect antibodies without functional correlation to antiviral efficacy. Thus, to be able to provide CP for efficient therapeutic applications, multiple analytical approaches are required to detect antibody titers and determine their neutralizing potential before plasma donations are conducted. However, the identification of suitable CP donors does not automatically allow for determining the time span of donor suitability.

In the present retrospective study, we analyzed 189 CP donations of 66 different CP donors (2–12 sequential donations) from two independent Austrian plasma-collecting institutions. CP was collected during the pre-vaccination era (April 2020–February 2021) and was analyzed for anti-SARS-CoV-2 anti-S IgG levels with respect to the factors sex, age, ABO blood group and severity of the COVID-19 disease course. The main objective of this study was to determine the optimal CP donation time span and thus donor suitability using the first IgG ratio value of a putative CP donor measured. Furthermore, we investigated whether the widely applied semi-quantitative IgG ratio value correlates with in vitro neutralizing antibody activity.

## 2. Materials and Methods

### 2.1. Ethical Statement

Human plasma collection was done according to the standards of the Association for the Advancement of Blood & Biotherapies’ guide *Apheresis: Principles and practice* [24] and the *Guide to the preparation, use and quality assurance of blood components* from the European Directorate for the Quality of Medicines and HealthCare [25]. All donors signed informed consent on the use of plasma donation intended to be applied as a therapeutic application for COVID-19 patients and on the use of leftover material for research purposes. The work described has been carried out in accordance with the 1964 Helsinki Declaration and its later amendments or comparable ethical standards. COVID-19 CP and samples thereof were processed anonymously to protect the privacy of each donor.

### 2.2. Plasma Donor Selection

COVID-19 CP was collected between April 2020 and February 2021 by two independent institutions: by the Blood Services of the Austrian Red Cross Vienna, Lower Austria and Burgenland, Austria and the Transfusion Department of the University Hospital Salzburg, Austria. All CP-donors were aged between 18 and 68 and fully recovered of RT-qPCR and SARS-CoV-2 anti-N antibody screening-confirmed COVID-19 for at least 4 weeks. No COVID19-related sequelae were reported at the time of CP donation. Female donors with a history of previous pregnancies were tested for HNA and HLA antibodies using Luminex microbeads technology according to manufacturer’s instructions (Luminex Corporation, Austin, TX, USA). Furthermore, CP donors were selected according to their ABO-blood group, which had to match with the blood groups of the patients treated. A pre-donation examination and clinical evaluation was performed at least one day prior to the first plasma donation to exclude potential contraindications. In the course of this health screening, blood samples were screened serologically and molecular biologically for HIV1/2, HAV, HBV, HCV, PB19 and syphilis as a part of the routine work-up of each plasma donation. Furthermore, a full blood count was done and levels of the inflammatory markers neopterin or C-reactive protein (CRP) were determined. In addition, plasma protein, immunoglobulins and creatinine were measured. Providing negative screening for the infectious disease parameters tested, results within the normal range for inflammatory markers, plasma protein, immunoglobulins, creatinine and full blood count and a SARS-CoV-2 anti-S IgG ratio of ≥2, donors were admitted to COVID-19 CP donation.

### 2.3. Plasma Collection

Using the Trima Accel cell separator (Terumo BCT, Lakewood, CO, USA), a maximum volume of 650 mL of COVID-19 CP was collected in the course of a donation event. Plasma pathogen reduction was performed applying the INTERCEPT blood system (Cerus Corporation Europe BV, Amersfoort, The Netherlands) according to manufacturer’s instructions. The collected CP was stored at ≤−25 °C until further usage.

### 2.4. Anti-SARS-CoV-2 Serological Screening

For SARS-CoV-2 anti-N donor screening, the Elecsys Anti-SARS-CoV-2 total antibody electrochemiluminiscence immunoassay (ECLIA, Roche Diagnostics, Basel, Switzerland) was applied using a cobas8000-e801 device (Roche Diagnostics) according to manufacturer’s instructions.

The SARS-CoV-2 testing of collected CP was performed with the semi-quantitative Euroimmun SARS-CoV-2 IgG ELISA (Euroimmun, Lübeck, Germany) (EI IgG ELISA) using an Euroimmun Analyzer I as described in Hoepler et al. [26]. In brief, this assay uses a recombinant spike protein as target, and testing was conducted according to the manufacturer’s instructions and with serial dilutions of donor plasma. Results were expressed as IgG ratio values. Ratios were automatically calculated by the Euroimmun Analyzer I platform according to manufacturer’s instructions by dividing the optical densities of the sample by those of an internal calibrator, which is provided with the test kit.

To determine in vitro functionality of SARS-CoV-2 anti-S antibodies detected, a SARS-CoV-2 Surrogate Virus Neutralization Test (sVNT) (GenScript, Piscataway Township, NJ, USA) was conducted. In brief, this ELISA-based assay screens for antibodies against SARS-CoV-2 being able to inhibit the protein-protein interaction between a horseradish-peroxidase-conjugated recombinant viral receptor binding domain (RBD) protein and the human angiotensin converted enzyme 2 (ACE-2). Results are expressed as percent signal inhibition (= net OD450 sample value/OD value of negative control × 100). As recommended by the manufacturer, signal inhibition rates ≥30% are considered as positive, indicating functional neutralizing antibodies against SARS-CoV-2, values <30% are considered as negative, indicating inadequate RBD-ACE-2 inhibition and thus insufficient antibody functionality.

### 2.5. Statistical Analysis

For the descriptive analysis of the data, correlations were calculated using Spearman‘s correlation coefficient. Calculations were conducted using the statistical software R (R-core team, Vienna, Austria, version 4.1.3) [27] or GraphPad Prism software (Dotmatics, Boston, MA, USA, version 9). For assessing statistical significance of differences between different ABO blood groups, a nonparametric ANOVA test was used. For the comparisons between two groups (e.g., male versus female) Wilcoxon-Mann-Whitney test was applied. A time-to-event analysis (i.e., Kaplan Meier curves, Cox PH model) was performed to analyze the impact of baseline levels on the probability of a drop of the anti-S IgG ELISA ratio value below three. For this purpose, the R package survival [28] was used as well as survminer for the graphical representation of the results [29]. For all hypothesis tests, the two-sided level of 5 percent was used.

## 3. Results

### 3.1. Study Cohort

As summarized in Table 1, the cohort of COVID-19 CP donors investigated in this retrospective study consisted of 57 male (= 86.4%) and 9 female (= 13.6%) donors, who in sum donated 189 CP products (median number of CP donations: 3, range 2–12 donations). The median age of donors was 43 years (range: 18–68 years). The distribution of ABO blood groups among CP donors reflects the physiological ABO blood group distribution in Austria (according to the Austrian Red Cross, https://www.roteskreuz.at/blutspenden/wissenswertes-zum-blut?gclid=EAIaIQobChMIj_2wz4rm9QIVwwyLCh0-4wjZEAAYASAAEgI0s_D_BwE, accessed on 10 August 2022) and is as follows: blood group A (*n* = 28, 42.4%), B (*n* = 12, 18.2%), AB (*n* = 5, 7.6%) and 0 (*n* = 21, 31.8%).

### 3.2. SARS-CoV-2 Anti-S Antibody Levels Are Independent of Age, Sex, ABO Blood Group and Disease Course

**Figure 1:**
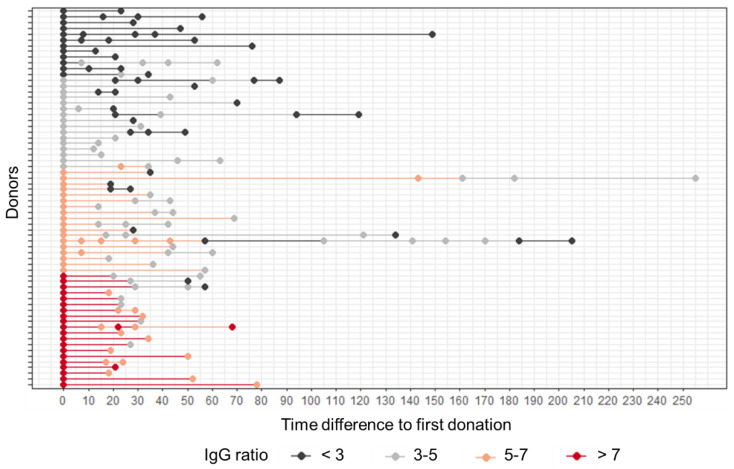
SARS-CoV-2 anti-S antibody levels of CP donors are declining over time. Data shown are anti-S IgG ratio values grouped as indicated for each plasma donation. Day 0 represents the first plasma donation, all further donations are depicted as time difference to the first donation, given in days.

CP donations took place 52 days (median value, range 33–93 days) after convalescence of a RT-qPCR and SARS-CoV-2 anti-N antibody screening confirmed SARS-CoV-2 infection. SARS-CoV-2 anti-S IgG kinetics was monitored for each plasma donation of each individual donor. It is important to note that for the time span investigated, no vaccination was available and thus only infection-acquired antibody levels were investigated. We observed varying levels of SARS-CoV-2 anti-S IgG antibody ratios for the first plasma donation (Figure 1): 12 donors showed an anti-S IgG ratio < 3, 16 showed IgG ratio values between 3 and 5, 18 donors had anti-S IgG ratio values between 5–7 and 20 CP donors showed an IgG ratio value > 7. As expected, we found declining SARS-CoV-2 anti-S antibody levels over time for the majority of CP donors (*n* = 42). Eighteen donors showed rather stable anti-S levels for the time span observed, while six donors showed rising and falling anti-S antibody levels (Figure 1).

**Figure 2:**
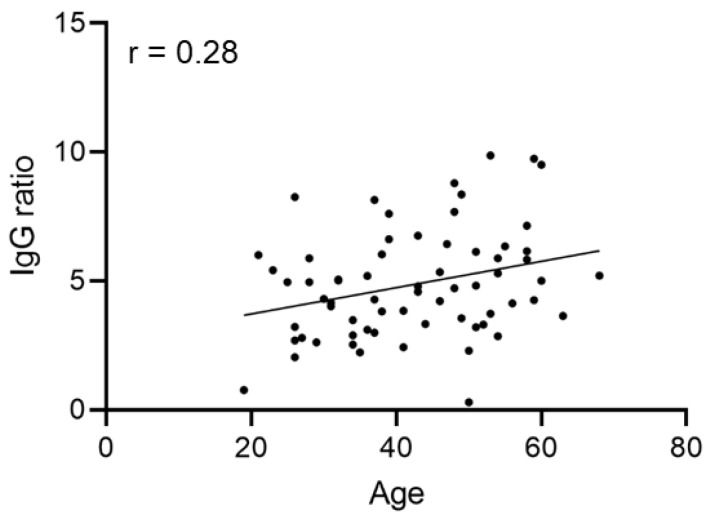
Donor age (given in years) does not correlate with the level of IgG ratio measured at the first CP donation (Spearman’s r = 0.28).

We next asked whether there is a correlation between IgG ratio values and the parameters sex, ABO blood group, age or disease course. A significant correlation between donor age and IgG ratio value of the first plasma donation (Figure 2) could not be found.

**Figure 3:**
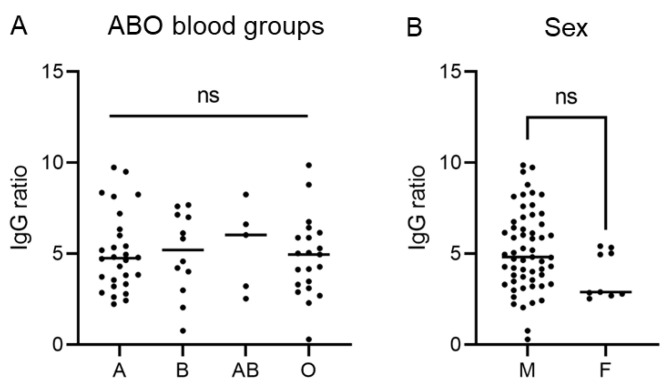
SARS-CoV-2 IgG anti-S ratio levels are independent of ABO blood group (**A**) and sex (**B**), male (M), female (F).

Furthermore, we did not observe significant differences between different ABO blood groups (Figure 3A) or male and female donors (Figure 3B).

**Figure 4:**
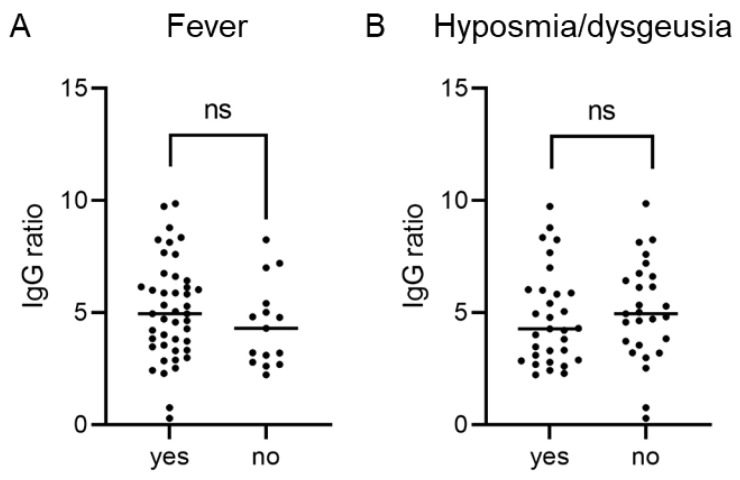
SARS-CoV-2 IgG anti-S ratio levels are independent of a COVID-19 disease course with or without fever (**A**) and with or without hyposmia and/or dysgeusia (**B**).

For 59 CP donors, data regarding the COVID-19 disease course were available. Forty-four CP donors reported a feverish course of the disease, 31 reported to have experienced hyposmia and/or dysgeusia. Comparing symptomatic and asymptomatic disease courses with respect to fever and hyposmia/dysgeusia using the Wilcoxon-Mann-Whitney test showed no significant difference regarding the level of SARS-CoV-2 anti-S IgG ratio values (Figure 4A,B).

### 3.3. SARS-CoV-2 Anti-S IgG Ratio Values Correlate with Antibody Functionality

**Figure 5:**
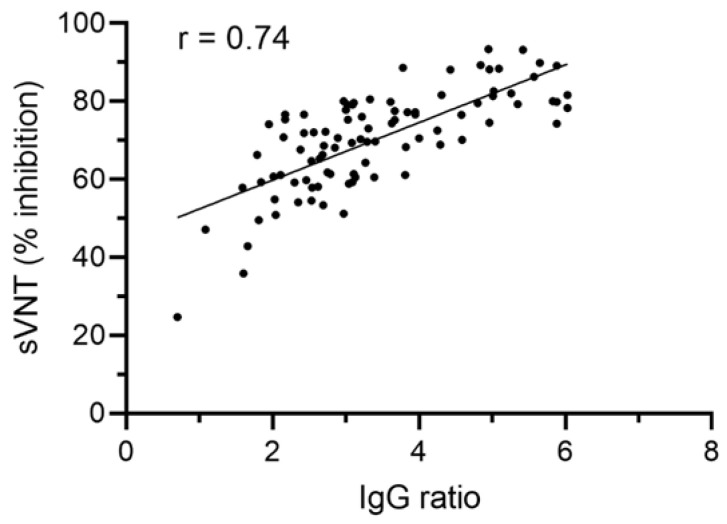
SARS-CoV-2 anti-S IgG ratio values correlate with in vitro antibody functionality. Spearman correlation analysis of 92 different CP donations indicates a significant correlation between SARS-CoV-2 anti-S IgG ratio values and in vitro antibody functionality detected by a surrogate virus neutralization test (sVNT). Spearman’s r = 0.74, *p* < 0.001.

We next asked whether the semi-quantitative IgG ratio value correlates with antibody functionality. Thus, we performed a SARS-CoV-2 surrogate virus neutralization test with a subset of 92 different CP donations of 24 individual donors. Our data show a significant correlation (Spearman’s r = 0.74, *p* < 0.001) between IgG ratio values and in vitro antibody functionality: CP samples with high IgG ratio values showed higher antibody functionality compared to CP with lower IgG ratio values (Figure 5).

### 3.4. The Level of SARS-CoV-2 Anti-S Antibody Allows Determining the Time Span of CP Donor Suitability

Our data indicate that SARS-CoV-2 anti-S antibody levels of CP donors are independent of age, sex, ABO blood group and disease course, pointing to strong donor variability. This makes the identification of suitable donors and especially the determination of the optimal donation time span for efficient CP a major challenge. We therefore investigated whether it is possible to predict the time span of CP donor suitability using the initial SARS-CoV-2 anti-S antibody ratio value of a putative CP donor. In order to do so, we defined an anti-S IgG ratio value of 3 as the minimum value, which has to be reached for suitable COVID-19 CP donation, which, according to our correlation analysis, would account for >60% in vitro antibody functionality (Figure 5).

**Figure 6:**
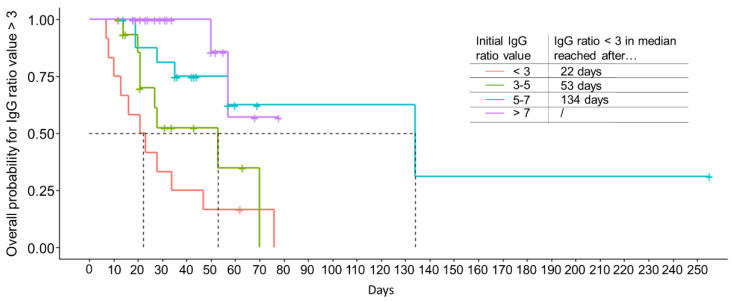
High SARS-CoV-2 anti-S IgG ratio values allow longer CP donation periods. Time-to-event analysis for four different groups of IgG ratio values: <3 (*n* = 12), 3–5 (*n* = 16), 5–7 (*n* = 18) and >7 (*n* = 20) was conducted. Data shown are overall probabilities for a drop of the IgG ratio value below the value of 3 over time given in days (median values are indicated by dashed lines), log-rank *p* < 0.0001.

Using a Cox model analysis, we could show that the individual level of SARS-CoV-2 anti-S IgG of the initial CP donation has a significant influence (hazard ratio 0.6, *p* < 0.0001) on anti-S antibody kinetics over time. This indicates that individuals with higher initial IgG ratio values show a lower risk of reaching an IgG ratio value below 3 for the time span observed. As a next step, a stratified Kaplan-Meier analysis was performed to calculate the overall probability for a particular initial anti-S IgG ratio value to drop below a value of 3 over time. As shown in Figure 6, our analysis revealed, that an initial IgG ratio value between 3 and 5 dropped below 3 after a median of 53 days and ratio values between 5 and 7 dropped below 3 after a median of 134 days. We could not determine such a point in time for initial IgG ratio values > 7, since there were only a few drops below the value of 3 within the time span observed (80 days after the first CP donation). In addition, the initial IgG ratio values < 3 were included in our analysis, since in some cases the IgG ratio value increased over the time span observed. For this group, the analysis revealed that in median after 22 days a drop to the minimum ratio value of 3 is reached again. Our data clearly indicate that higher IgG ratio values allow for a longer CP donation period (log rank test *p* < 0.0001), in line with the above-mentioned results of the Cox model using the raw uncategorized IgG ratio values as the explanatory variable.

## 4. Discussion

CP application is one of the first therapy options in epidemics, as it becomes available as soon as there are convalescent individuals. Even though it has been in use for numerous epidemic outbreaks over the last century, its therapeutic efficacy is still under debate. Several studies from China were among the first reporting the application of CP to fight COVID-19 [19,30,31,32]. The first reports indicated an effective and safe therapy form, leading to reduced SARS-CoV-2 viral loads and improving clinical outcomes compared to non-CP-treated cases of COVID-19. In contrast, other studies indicate no significant improvements regarding COVID-19 disease course or mortality rate [21,33]. A third group of studies reports ameliorations only for specific patient cohorts: Joyner et al. reported that patients, who were not mechanically ventilated, show a lower mortality rate when treated with CP [17]. Another study reported a lower mortality rate for COVID-19 patients with hematological cancers after CP transfusion [34]. Based on current knowledge, CP therapy appears to be safe. However, it remains unknown which minimal virus-neutralizing capabilities a CP has to provide for the improvement of clinical symptoms. A recent review summarizes the controversial findings in the field and concludes that the absence of a benefit of CP therapy may be explained by insufficient therapeutic doses applied, thus highlighting the importance of appropriate IgG levels and presence of neutralizing antibodies in CP [35].

In this retrospective study, we found that infection-acquired SARS-CoV-2 antibody levels are heterogeneous among CP donors and generally decline over time. The level of IgG ratio values observed does not dependent on age, sex, ABO blood group or previous COVID-19 disease course. These findings are in line with other studies that previously demonstrated no significant correlation between SARS-CoV-2 antibody levels and sex, age and ABO blood group [36,37,38,39,40,41,42].

Regarding sex, it should be considered that there is a gender imbalance in our present study (57 male and 9 female CP donors). This can be explained by previous pregnancies, which elevate the risk for HNA/HLA antibodies in female donors. Therefore, male individuals are often preferred as blood/CP donors. Concerning age, there are studies describing age-associated differences regarding SARS-CoV-2 antibody levels: Yang et al. investigated a wide range of patient age groups (1–102 years) and showed that COVID-19 patients aged 19–30 years exhibited the lowest IgG levels [43]. This contradictory finding might be explained by utilizing the investigation of different study sample sizes and age groups, as only individuals aged between 18 and 68 were included in our study.

A study conducted by Klein et al. reported that severe disease courses, which were associated with hospitalization, resulted in elevated antibody responses [44]. Others reported similar results for comparisons of hospitalized individuals and outpatients [45,46,47]. In contrast, our study revealed no significant difference regarding IgG ratio values for disease courses with or without fever or hyposmia/dysgeusia. This may be explained by the fact that only one CP donor in our study was hospitalized due to COVID-19, pointing to a generally milder disease progression among our study cohort.

In order to be able to predict CP donor suitability over time by using the IgG ratio value, we first defined the IgG ratio value 3 as the minimum value to be reached for CP donation suitability. This value was chosen as according to our analysis; it refers to >60% in vitro antibody functionality. We further grouped our study cohort according to the initial IgG value measured into four subgroups: IgG ratio value < 3, IgG ratio value between 3 and 5, IgG ratio value between 5 and 7 and IgG ratio value > 7. Our data indicate that individuals with higher initial IgG ratio values show a lower risk of reaching the limit of IgG ratio value below 3 over time. According to our findings, this allows a longer CP donation period compared to donors with lower initial IgG values. Our analysis further allowed the median prediction of donor suitability in days: initial IgG ratio values between 3 and 5 dropped below a value of 3 in median after 53 days, ratio values between 5 and 7 dropped below the limit of 3 in median after 134 days.

Even though CP therapy efficacy is still under debate, there are suggestions for successful CP application. Klassen et al. suggested the use of CP containing specific, neutralizing antibodies prophylactically or at an early point in time to obtain therapeutic benefit [23]. Furthermore, it becomes evident that sufficient neutralizing antibody levels present in a plasma product are essential for successful therapeutic measures [35]. Additionally, the European commission directorate for health and food safety also requests blood service centers in Europe to issue CP with the highest antibody titers available [48]. However, the directorate also states that there is no international standardized unit for the term “titer”. This makes the comparison of CP from different producers difficult [48]. In our study, we combined two conventional assay approaches, a semi-quantitative ELISA and an ELISA-based in vitro functionality screening. Our data show that semi-quantitative anti-S IgG ratio values significantly correlate with the in vitro inhibition rate of the RBD-ACE2 interaction, thus indicating the level of antibody functionality. This is an important finding, which may help CP producing institutions to choose the optimal donor, donation period and thus plasma product for treatment of COVID-19 patients.

Taking into account that SARS-CoV-2 variants might emerge, which may acquire resistance to currently available monoclonal antibodies, COVID-19 CP should be considered as first-line therapy, which is efficient when applied early with adequate neutralizing antibody activity present.

Our retrospective study has some limitations: first, even though two Austrian plasma-collecting institutions were included, the study cohort (*n* = 66 donors) is rather small. This is because we observed the developmental course of IgG ratio values over time. Thus, donors who donated CP just once were excluded from this study. Furthermore, donors donated CP according to therapeutic/medical requirements of the patients treated. Therefore, the donation period and frequency observed are rather heterogeneous. Finally, since our retrospective study regards CP from the pre-vaccination era, we cannot exclude different findings for COVID-19 CP from vaccinated individuals.

## 5. Conclusions

In conclusion, our data reveal that the frequently used semi-quantitative anti-SARS-CoV2 IgG ratio values directly correlate with specific antibody functionality in vitro. Furthermore, our analysis shows that the IgG ratio value allows for predicting the median time span for CP donor suitability: While CP donors with a higher IgG ratio value (between 5 and 7) were suitable for CP donation for a median of 134 days, donors with IgG ratio values between 3 and 5 were suitable for a median of 53 days. Our findings will support plasma collection centers and clinicians to choose suitable CP donors and predict the optimal time span for CP donation, thus leading to efficient COVID-19 CP products for clinical applications.

## Figures and Tables

**Table 1:** CP donor study cohort.

Total Number of Participating Donors/Donations	66/189
Sex	
male	57
female	9
Age	
19–25 years	4
26–35 years	17
36–45 years	15
46–55 years	20
56+ years	10
Median age: 43 years	
ABO blood group	
A	28
B	12
AB	5
0	21

## Data Availability

The data presented are not publicly available to protect privacy of CP donors.
